# Progress in Respiratory Gene Therapy

**DOI:** 10.1089/hum.2022.172

**Published:** 2022-09-01

**Authors:** Gerry McLachlan, Eric W.F.W. Alton, A. Christopher Boyd, Nora K. Clarke, Jane C. Davies, Deborah R. Gill, Uta Griesenbach, Jack W. Hickmott, Stephen C. Hyde, Kamran M. Miah, Claudia Juarez Molina

**Affiliations:** 1 The Roslin Institute & R(D)SVS, Easter Bush Campus, University of Edinburgh, Edinburgh, United Kingdom; 2 UK Respiratory Gene Therapy Consortium, United Kingdom; 3 Gene Therapy Group, National Heart and Lung Institute, Imperial College London, London, United Kingdom; 4 Centre for Genomic and Experimental Medicine, IGMM, University of Edinburgh, Edinburgh, United Kingdom; 5 Gene Medicine Group, Radcliffe Department of Medicine (NDCLS), University of Oxford, Oxford, United Kingdom

**Keywords:** Respiratory, Gene therapy vectors, AAV, Lentivirus, Gene Editing, Lung Disease

## Abstract

The prospect of gene therapy for inherited and acquired respiratory disease has energized the research community since the 1980s, with cystic fibrosis, as a monogenic disorder, driving early efforts to develop effective strategies. The fact that there are still no approved gene therapy products for the lung, despite many early phase clinical trials, illustrates the scale of the challenge: in the 1990s, first generation non-viral and viral vector systems demonstrated proof-of-concept but low efficacy. Since then, there has been steady progress towards improved vectors with the capacity to overcome at least some of the formidable barriers presented by the lung. In addition, the inclusion of features such as codon optimisation and promoters providing long-term expression have improved the expression characteristics of therapeutic transgenes. Early approaches were based on gene addition, where a new DNA copy of a gene is introduced to complement a genetic mutation: however, the advent of RNA-based products that can directly express a therapeutic protein or manipulate gene expression, together with the expanding range of tools for gene editing, has stimulated the development of alternative approaches.

This review discusses the range of vector systems being evaluated for lung delivery; the variety of cargoes they deliver, including DNA, antisense oligonucleotides, mRNA, siRNA and peptide nucleic acids; and exemplifies progress in selected respiratory disease indications.

## Introduction

Respiratory diseases are amongst the leading causes of death globally with numbers rising significantly since 1990^1^. Few effective treatments exist for many indications, often as a result of poor understanding of the disease aetiology. This unmet need has stimulated efforts to develop gene therapeutic approaches for a wide range of genetic, acquired and infectious diseases of the lung. The current era of genomics and transcriptomics has identified causative genes for monogenetic diseases, and a growing understanding of genetic pathways associated with polygenic or acquired conditions. As a result, new potential targets for therapeutic intervention are constantly emerging. This, together with an increasing understanding of stem cells, the availability of induced pluripotent stem (iPS) cells, the ability to direct their differentiation to specific lineages and the rapidly expanding toolbox available for gene replacement/editing is fuelling renewed efforts to develop new gene and cell therapy-based approaches.

The early days of gene therapy coincided with the identification and cloning of the gene for cystic fibrosis (CF)^2^. This, together with the notion that target epithelial cells in the lung were topically accessible through the airway, meant that CF came to be regarded as the prime candidate for gene therapy development for Mendelian conditions. There was significant activity in the 1990s using vectors based on adenovirus, (Ad), Adeno associated virus (AAV) and non-viral (synthetic) formulations, culminating in a series of early phase clinical trials in CF patients^3,4^. There were no significant safety concerns and proof of concept in terms of vector mRNA expression and transient changes was widely achieved: however, the low efficacy and transient nature of transgene expression was disappointing. It should be noted, however, that these primarily safety studies did not assess clinically relevant efficacy endpoints.

## Challenges and Barriers

Initial preconceptions were that delivery to the lung topically either via bronchoscopy or inhalation, would be straightforward. Unfortunately, delivery proved to be a considerable challenge due to factors such as the complex structure and function of the lung, its large surface area, and the physical barriers that have evolved to protect the lung from inhaled particulates (such as gene therapy formulations) and pathogens^5^. Various alternative non-topical delivery strategies have been used in attempts to achieve transgene expression in the lung/interstitium. Intrapleural delivery of vectors for a secreted transgene product such as alpha-1 antitrypsin AAT has the potential advantage of achieving both local expression via transduction of the pleural mesothelial cells and systemic expression through transduction of hepatocytes after lymphatic circulation of vector to the liver^6^. However, systemic administration, being relatively inefficient and leading predominantly to lung endothelial delivery^7^, will not be a focus of this review.

Physical barriers that prevent material reaching the airway epithelial cells include the airway surface liquid and glycocalyx in combination with mucociliary clearance^8,9^. In many cases the receptors used by viral vectors are not exposed at the apical surface, but instead reside at the basolateral surface obscured by tight junctions^10^. Strategies to promote accessibility to receptors on the basolateral surface have included forms of chemical preconditioning that transiently open the tight junctions^10^. There is also the additional barrier of viscous mucus in the airways of CF lungs which is not only difficult to clear from the airways but can also trap inhaled particles and inhibit gene transfer^11^. An important, but not lung-specific, barrier that relates more to the delivery of viral vectors is the presence of pre-existing or acquired immunity that can prevent initial or repeat transduction^12,13^. Finally, a non-biological challenge is that due to the lung’s large surface area, the manufacturing scale-up required to generate sufficient amounts of gene therapy vector for in vivo delivery significantly increases the cost of vector production.

## Gene Addition

### Viral Vectors

#### Adeno associated virus (AAV) vectors

AAV vectors have become the vector of choice for many gene therapy applications due to their perceived low risk of pathogenicity, the large range of serotypes that allow transduction of a broad range of target organs, the lack of a strong immune response to some of the commonly used vector serotypes in vivo, and the duration of expression in non-proliferating cells. One of the most important limitations of AAV vectors is the small genome packaging capacity of ~4.6 kb which limits cargo size. AAV vector’s capacity for delivery to the lung has been extensively evaluated in both pre-clinical and clinical studies over more than 20 years^14^. Early studies of clinical lung delivery were focused on CF and attempted to target conducting airway epithelial cells using AAV serotype 2 vectors. Between 1999 and 2007, several Phase 1 trials^15^ demonstrated the excellent safety profile of rAAV but failed to meet their efficacy endpoints. Transgene mRNA levels were low because packaging constraints forced these vectors to use the AAV Inverted Terminal Repeat (ITR) promoter to drive CFTR expression. Compounding this, transduction efficiency was also low because the receptor required for AAV2 is localised on the basolateral membrane of target cells^16^.

Numerous efforts have been made to alter the tropism of AAV vectors by generating synthetic serotypes using techniques such as directed evolution^17,18^, capsid shuffling^19^, peptide display^20^ or modification of surface topology of variable loop regions^21^. This has led to the development of AAV serotypes AAV2.5T and 4D-A101 both of which demonstrate apical transduction of airway epithelial cells. AAV2.5T has been shown to use sialic acid for internalisation and exhibits several-fold improvement in apical targeting compared to the native serotype^22^. The mechanism for receptor attachment of 4D-A101 has not yet been elucidated: however, there is welcome evidence that it exhibits resistance to pre-existing anti-AAV human nAbs in vitro^18,23^.

Strategies have also been developed in an attempt to overcome the limitations of packaging capacity. One such strategy involves the use of a dual AAV vector system, with each vector carrying parts of the larger gene designed to recombine in vivo. Although there is evidence that the strategy is effective in mouse lungs with high vector doses^24^, it seems unlikely that this will be successfully translated to the clinic due to the combination of complexity of production, delivery and frequency of homologous recombination in the differentiated airway epithelial cells. An alternative approach is a dual vector system where one vector carries the transgene flanked by the terminal repeats of the piggyback (PB) transposon, and the other carries the PB transposase to promote integration of the transgene cassette when both vectors are expressed in the same cell^25^. Although the vector carrying PB-flanked transgene was shown to transduce the airway epithelium in CF knockout pigs, integration by dual vector co-infection has not yet been achieved in this model.

Another approach to overcome rAAV packaging constraints is the generation of parvovirus chimeras using human and gorilla bocaviruses (HBoV, GboV) that naturally transduce airway epithelia via the apical surface. rAAV/HBoV and rAAV/GBoV can package AAV genomes that are too large to be packaged by conventional rAAV and have been shown to effectively transduce primary human airway epithelia cultures, primary lung organoids, and ferret lungs^26,27^. The low production titres of these chimeric vectors achieved in early studies have stimulated the development of improved production protocols to generate sufficient high titre vector for clinical application (reviewed by Vu & McCray 2020) ^28^.

The use of AAV vectors in the lung for chronic diseases where repeat administration is likely to be required as a a result of cell turnover in the lung, is currently limited by the induction of potent neutralising antibodies generated in response to the original vector administration^29^. Strategies to modulate the immune system, such as B cell depletion with anti-CD20 antibody or the use of rapamycin to modulate T-cell responses, have shown promise in minimising immune responses to capsid or transgene^30,31^. It has been suggested that encapsulating AAV vectors in exosomes as a mechanism of immune evasion may also be a useful strategy^32^. It should be noted that to date these immune modulation strategies have not been demonstrated to be effective in the context of gene delivery to the lung.

The long-held contention that AAV vectors are non-pathogenic has recently been called into question by an increasing body of evidence that they are capable of fragmentary integration into the host cell genome in animals and humans^33^ and can cause hepatotoxicity, thrombotic microangiopathy and neurotoxicity. AAV vector-mediated insertional mutagenesis resulting in hepatocellular carcinoma has been observed at the mouse Rian locus^34^. Clonal expansion of hepatocytes has also been reported in a canine model following recombinant AAV delivery although tumour formation was not observed^35^. However, AAV vector integration events associated with tumours have only been observed in the livers of rodent species, and generally seem to be associated with high doses of vector. The FDA Cellular, Tissue, and Gene Therapies Advisory Committee (CTGTAC) Meeting #70 of September 2-3, 2021 specifically addressed the emerging toxicity risks of rAAV for gene therapy, and provides a comprehensive summary of the associated risks together with FDA recommendations.^36^

### Lentiviral vectors

The attraction of lentiviral vectors for gene therapy applications comes from their ability to transduce non-dividing cells and achieve persistent transgene expression via integration into the host genome. Following the genotoxicity events that resulted in malignancies due to insertional mutagenesis in the SCID-X1/gamma-retrovirus clinical trials there has been a switch from gammaretroviral to safety-enhanced lentiviral vectors. Since 2008, hundreds of patients have received therapies that have involved ex vivo treatment of hematopoetic stem cells (HSCs) with lentiviral vectors (reviewed Tucci et al 2022^37^). The safety concerns over the potential for genotoxicity have been mitigated by the development of self inactivating (SIN) vectors in which the promoter/enhancer from the lentiviral Long Terminal Repeat sequences has been deleted. It has also been established that unlike gammaretroviral vectors, lentiviral vectors do not preferentially integrate into transcriptional start sites and so intrinsically have a significantly better safety profile^37^. Vectors based on human^38,39^ feline^40–42^ and simian^43–45^ immunodeficiency viruses (HIV, FIV, SIV) have been shown to transduce airway epithelial cells with encouraging safety profiles in a number of species. The typical lentiviral vector pseudotype, derived from the envelope glycoprotein of vesicular stomatitis virus (VSV-G), mediates poor airway transduction because the cognate receptors are not apically localised^46^. To address this, studies of HIV.VSV-G vectors have used preconditioning agents such as EGTA, perfluorocarbons and the mild detergent LPC to transiently open tight junctions and facilitate access ^47,48^, but translation of this strategy to the clinic is doubtful. Different pseudotyping strategies have retargeted FIV and SIV vectors to the apical membrane using the baculovirus surface glycoprotein GP-64 (rFIV-GP64) or the Sendai virus hemagglutinin-neuraminidase (HN) and fusion (F) glycoproteins (rSIV-F/HN). Both of these vectors mediate persistent transgene expression in airway cells in a variety of preclinical models in vitro and in vivo, and more importantly, are able to be administered repeatedly without significant loss of transgene expression^41,44^. Envelope glycoprotein pseudotypes derived from respiratory syncytial virus (RSV)^49^, Marburg and Ebola virus^50,51^, influenza HA-M2^52^, severe acute respiratory syndrome (SARS) spike protein^53^, and Jaagsiekte sheep retrovirus (JSRV)^54^ have also been evaluated for mediating apical entry to airway epithelial cells. However, the use of some pseudotypes, notably GP64, can result in low production titres. Circumvention of this by using producer cell lines has been reported: a stable HEK273 cell line expressing higher levels of the GP64 than can be obtained in transient transfection systems together with improved vector purification has allowed the production of high titre vector^55^.

The high packaging capacity of lentiviruses also make them suitable for the delivery of larger cargoes, including Cas protein and single or multiple guide RNAs for gene editing from a single vector^56^, which suggests a multiplex vector approach to regulate gene expression may be feasible.

### Helper-dependent adenoviral (HD-Ad) vectors

HD-Ad vectors are replication incompetent constructs in which non-essential viral coding sequences have been deleted. As a result, such vectors are less immunogenic than conventional Ad vectors^57^. While multiple serotypes are available, serotype 5 is most frequently used in the lung, effectively transducing airway basal cells in vivo in mouse and pig models^58^. There is evidence that one readministration of HD-Ad to the mouse lung is possible with a relatively low dose^59^: however, single re-administration at the high dose required to achieve sufficient efficacy requires transient immunosuppression^60^.

Hd-Ad vectors express transiently and have a very large packaging capacity (~36 kb), which makes them an attractive option for gene editing, since they can accommodate Cas protein, gRNA, and donor DNA within a single vector. Transient expression of Cas9 in particular reduces the risk of an immune response that eliminates the gene modified cells^61–63^. To date, however, this has only been demonstrated in airway cells in vitro.

### Herpes simplex virus type 1: HSV-1

HSV possesses numerous biological features that make it attractive as a gene delivery vehicle for the nervous system and other tissues. The virus possesses a broad host range and is able to transduce nondividing cells, notably neurons, and dividing cells at extremely high efficiencies. The large capacity of the viral genome (152 kb), and the fact that many viral genes can be removed as contiguous segments without dramatically affecting virus production, have enabled the incorporation of large or multiple transgenes, making it a preferred vector for expression of multiple gene products or gene libraries (reviewed by Goins et al 2020^64^). Although better known for delivery to the central nervous system or for oncolytic approaches to tumour therapy, HSV-1 vectors are also being developed for gene delivery to the lung. Vectors expressing two transgene copies have been reported in conference proceedings to exhibit good gene transfer and the capacity for repeat administration in the lung due to the lack of a significant immune response to the vector^65–67^. The lack of peer reviewed publications on these vectors to date, make it more difficult to fully assess their potential.

### Non-viral/nanoparticle Vectors

There are many attractions of non-viral vectors for the delivery of nucleic acids. They have an almost unlimited packaging capacity and are generally more biocompatible and hence safer than viral vectors. In particular, the risks of insertional mutagenesis are vanishingly small. However, efficient delivery of non-viral vectors to lung cells has proved to be extremely challenging, and they typically lack cell-type specificity. There have been multiple attempts to improve the specificity of cell targeting and enhance expression through complexation/conjugation manoeuvres with lipid, peptides or polymers to form nanoparticles^68–70^. Between 1995 and 2004 a number of clinical trials of non-viral gene therapy, mostly for cystic fibrosis, using plasmid DNA and cationic liposomes or polymers, were performed (reviewed in Sondhi et al 2017^14^). Overall, the outcome was that the formulations had excellent safety profiles, but failed without exception to meet efficacy outcomes. However, it should be noted that most were Phase 1 studies with limited secondary outcomes of efficacy measuring for example transgene DNA, mRNA, and changes in epithelial chloride transport.

An exception was the Phase 2b randomised control trial (RCT) in CF patients of cationic liposome GL67A, in which inhaled delivery of an improved CpG-depleted plasmid DNA construct reached its clinically relevant primary outcome ^71^, an important proof of concept but not of sufficient magnitude to warrant immediate progression to Phase 3 since observed effect was mainly due to decline in lung function in placebo arm rather than improvement in response to treatment. This formulation also exemplified the reduced propensity of synthetic non-viral formulations to exhibit cytotoxicity or to elicit adverse immune responses confounding re-administration: multiply repeat dosing of GL67A complexes to the airways without loss of expression was demonstrated in the Phase 2b RCT and in other species^71–73^.

The emergence of RNA-based therapeutic approaches (mRNA, siRNA, miRNA, ASO) and gene editing technologies has renewed interest in transient and safer non-viral delivery. Several clinical trials of inhaled RNA therapy have been initiated including delivery of siRNAs for asthma (Excellair) and RSV infection (ALN-RSV01), full CFTR mRNA delivery for CF (MRT5005) and an antisense oligo also for CF (Eluforsen). However, no inhaled RNA therapeutic has yet been approved for clinical use (reviewed in Chow et al, 2020^74^). Product development of both Excellair and ALN-RSV01 was discontinued at the early phase clinical trial stage. Further discussion of MRT5005 and Eluforsen clinical trials can be found in the section on CF below.

## Gene Editing

The last decade has witnessed an explosion in technologies for precise gene editing that offers the potential to edit and repair mutated genes in vivo, insert new genes, or delete undesirable genes. Gene editing depends on sequence-specific, programmable nucleases: it began with zinc finger nucleases (ZFNs) and transcription activator-like effector nucleases (TALENs), but these have largely been superseded by the clustered regulatory interspaced short palindromic repeats (CRISPR) and CRISPR associated 9 nuclease (Cas9) systems and its derivatives which are simpler, more flexible and highly efficient tools for gene editing. The system utilises a guide RNA (gRNA) complementary to the target sequence near a protospacer adjacent motif (PAM) site. A PAM interaction domain of the Cas9 effector nuclease then mediates double strand cleavage. The DSB can be repaired by error prone non-homologous end joining (NHEJ) that typically results in frameshift or nonsense mutations, or by the less efficient but therapeutically more useful homology-directed repair (HDR) process if a donor repair template is provided. Preclinical studies in airway cells or lung organoids have shown promise^75–77^. However, the huge challenge for translation of HDR-based editing will be ensuring efficient delivery of the required components to sufficient numbers of cells in vivo, given that the frequency of editing may be low, for example in terminally differentiated airway epithelial cells where homologous recombination is minimal.

A number of viral vectors are being evaluated for delivery of CRISPR/Cas9 gene editing components to the lung. A recent report from the NIH Somatic Cell Genome Editing (SCGE) Consortium demonstrates the activity of a dual AAV5 vector system, one to deliver the Cas9 and one to deliver two gRNAs, following delivery to the mouse lung^78^. Editing of a lox-STOP-lox-Tomato reporter with efficiencies of ~20% was achieved in the large and small airways of mice. This editing was based on the use of two gRNAs targeted either side of a STOP cassette to delete it and hence relied on NHEJ rather than co-delivery of a template DNA for homology-directed repair that might be required to specifically correct a mutation. The system would still have to overcome the lack of ability to repeat administer for rAAV unless the vector was able to edit resident stem/progenitor cells.

More recently, base editing and prime editing strategies have been developed which confer higher specificity/efficiency and lower off-target activity than conventional gene editing. Base editing allows the precise editing of specific nucleotides without inducing a DSB, using a combination of gRNAs to target the relevant genomic site and base editors (BEs) to effect the desired change, such as cytidine BEs that convert C:G to T:A base pair and adenine BEs that convert A:T to G:C. The flexibility of base editing is restricted by the requirement for a PAM site at the target location. Prime editing removes the requirement of a PAM site and involves the use of a modified Cas9 that creates a single strand break and a prime-editing-extended guide RNA (pegRNA)-guided reverse transcriptase for reverse transcription of the pegRNA template to DNA. PEs are much larger than Cas9 and bring additional delivery challenges that will need to be addressed. The proof of concept for editing the *CFTR* gene in patient-derived cells or organoids using these technologies has been demonstrated^79,80^. However, the ability to translate these clinically will require efficient delivery systems and although some progress is being made towards this objective^81^ only time will tell whether these will prove clinically effective. Major questions for gene editing strategies for targeting the lung are (i) can formulations be devised that can treat diseases caused by any of a large number of mutations (such as CF)? (ii) can off-target events be reduced to an acceptably low level?

## Diseases

### Cystic Fibrosis

Cystic fibrosis (CF) is an autosomal recessive disorder which is relatively common in the Caucasian population with a prevalence of 7-8 per 100,000 in the US and EU^82^. The disease arises as a result of mutations in the *CFTR* gene, which encodes the CF transmembrane conductance regulator. CFTR functions as a chloride channel predominantly in the apical membrane of many epithelia, and has an important role in regulating fluid homeostasis and transporting ions across the epithelial barrier: in CF, however, the effects of CFTR deficit in the lung cause most morbidity. The development of CFTR modulators, in the form of small molecule potentiators or correctors that improve lung function, either alone or in combination, has had a transformative impact on a large proportion of CF patients^82,83^. Despite these effective new therapies for CF, there remains a cohort of patients who are either genotypically ineligible for modulator therapies or are unable to tolerate them: this justifies continuation of efforts to develop gene therapies agnostic to the mutation type a patient possesses. There is also the enticing possibility of combination therapies where the function of delivered CFTR is synergistically augmented by a modulator^84^. A range of approaches for CF gene therapy are being developed that exploit the expanding range of therapeutic options including gene addition and genome editing. A selection of the most promising candidates are described below.

#### CF Gene Therapy

##### Adeno-Associated Virus: AAV

After the initial wave of clinical trials for CF in the mid to late 1990s that mostly used Ad vectors or non-viral vectors^14^, AAV vectors have become the most favoured vectors for gene delivery to the lung. Factors in their popularity include the more favourable safety profile than Ad vectors which are highly immunogenic and evoke host immunity to the vector^85^, and higher efficiency than non-viral vectors. As discussed above, early trials with an AAV2 vector carrying the full length *CFTR* cDNA^86^ lacked efficacy, and drove the generation of vector serotypes that can successfully target the apical membrane of airway cells ^28^. The additional development of CFTR minigenes that retained functional chloride transport has provided sufficient space in vectors to include separate internal promoter and polyadenylation sequences^87^. Now, 15 years since the last AAV vector clinical trial for CF and even though the problems with repeat administration have yet to be solved, one clinical trial of an AAV vector 4D-710 is recruiting for a Phase 1/2 trial in adult CF patients^18,23^ and another is in the advanced preclinical development phase (SP-101). 4D-710 was identified by 4D Molecular Therapeutics through a process they have termed “Therapeutic Vector Evolution” that screened capsid variants using aerosol delivery in non-human primates (NHPs). The vector was well tolerated and showed resistance to pre-existing human antibodies in in vitro assays. The first patients in the 4D-710 clinical trial have recently been dosed. SP-101 (AV2.5T-SP183-hCFTRΔR) is composed of a novel capsid AAV2.5T optimized for efficient apical transduction of human airway epithelial (HAE) cultures and encodes a shortened CMVie promoter/enhancer that drives expression of a human *CFTR* minigene^17^. Both of these vectors use the *CFTR* sequence with deletion of a portion of the regulatory (R) domain (hCFTRΔR) to overcome packaging size restrictions. Another feature reported for SP-101 is that when used in combination with the drug doxorubicin in vitro in CF HAE cells, the level of functional CFTR-mediated chloride conductance increased in a dose-dependent manner^88^. This is in line with the documented ability of doxorubicin to enhance mRNA expression from AAV vectors^89^. It remains to be seen whether the improved apical transduction efficiency will be replicated in the clinical trial setting and lead to therapeutic levels of CFTR expression and, importantly, whether these engineered capsids will permit repeated administration of the vectors. Although existing AAV vectors do not have the capacity to carry the full CRISPR/Cas9 cargo, strategies to overcome this are being developed. These include the use of a smaller Cas9 variant, dual vectors where one carries the Cas9 protein and another the gRNA /donor template for HDR, or where the Cas9 N-terminal and C-terminal regions are encoded by separate vectors in a split Cas system^90,91^.

##### Helper-Dependent Adenovirus: HD-Ad

Helper Dependent (HD)-Ad was designed as a way of avoiding the adverse immune responses associated with adenoviral Ad vectors to confer persistent expression and retain the advantage of the large packaging capacity. HD-Ad vectors for airway expression, using control elements from the human cytokeratin 18 (K18) gene to drive expression of the CFTR transgene were developed for CF gene therapy and showed promise in preclinical studies. Prolonged gene expression and reduced inflammatory response was observed compared with Ad vectors. However, an immune response to the capsid proteins was still noted that blunted the efficacy of a second dose^59^. The transient use of cyclophosphamide around the time of delivery, when capsid proteins are predicted to be present, was able to block the production of neutralising antibodies and allow administration of a second dose that generated the same transduction efficiency as a single dose^60^. Of course, even transient immunosuppression may not be a practical option in CF patients with chronic bacterial lung infection, so despite some relatively promising preclinical data combining immunosuppression with HD-Ad vectors, this lung delivery strategy has not been trialled clinically.

The emergence and rapidly evolving field of gene editing has renewed interest in the use of HD-Ad vectors in the CF lung. Transduction of basal cells in the airways of piglets with HD-Ad vector has recently been demonstrated although the efficiency of basal cells targeting was not quantified in this study^58^. Having demonstrated efficient delivery of CRISPR/Cas9 plus CFTR donor template with an HD-Ad vector, and observed precise integration into the GGTA safe harbour site through HDR^61^, the authors speculate that delivering gene editing cargo in this way to basal cells, which possess stem/progenitor properties, may lead to long term gene correction^58,63^.

###### Lentivirus: LV

Lentiviral vectors have several features that make them an attractive platform for gene transfer to the lung. In particular, they readily transduce terminally differentiated cells in the airway epithelium, and with appropriate promoters, provide transgene expression for the lifetime of the cells. This is an important consideration for the treatment of chronic diseases such as CF where lifelong expression will be required. The first lentiviral vectors evaluated for CF gene therapy were HIV vectors with the typical VSV-G pseudotype^38,39,92,93^ which raised the previously discussed issue of basolateral receptors and the consequent need to open tight junctions to overcome this barrier. Pretreatment with lysophosphatidylcholine (LPC) in vivo permitted efficient and persistent gene expression in mice and provided evidence of recovery of CFTR function in CF mouse models^38^. Despite the promise, this strategy has not yet been tested clinically: this may in part relate to concerns about potential adverse effects of opening tight junctions in the diseased lungs of CF patients. An alternative approach has been to genetically modify the lentiviral envelope proteins by pseudotyping and thus re-target to the apical membrane. FIV pseudotyped with GP64 and SIV pseudotyped with Sendai F/HN have gained most traction in terms of progress towards translation. Importantly, both of these vectors have demonstrated the capacity for multiple repeat administration without significant loss of efficacy due to neutralising antibody responses^41,44^. The rFIV.GP64 vector has been shown to correct CF defects, including the anion transport defect, air surface liquid pH, and bacterial killing, after a single dose in CF pigs^42^ and is the basis for product SP-102 in the pipeline of Spirovant (https://spirovant.com/science/#pipeline). Preclinical studies with rSIV-F/HN in the murine lung have demonstrated that a single intranasal dose of the vector can generate transgene expression for the lifetime of the animals (at least 2 years), can achieve an average of ~15% transduction in the airway epithelium and can be repeatedly administered (daily or monthly) resulting in a cumulative dose-related increase in transgene expression^44^. In addition, the receptor used by the F/HN pseudotype for cell entry has been identified and shown to be present on target airway cells in human lung models^94^. Importantly, insertion site (IS) analysis found no evidence of bias in the distribution of integration sites. Although the F and HN proteins from murine Sendai virus and human parainfluenza virus (hPIV) 1 possess significant homology, hPIV1-specific antibodies had no impact on transduction in vivo when mice were pre-conditioned and then dosed with the rSIV-F/HN vector^45^. Overall the efficacy, low toxicity and IS profile together with the collaborative efforts of the UK Respiratory Gene Therapy Consortium, Boehringer Ingelheim (BI) and Oxford Biomedica have led to licensing of the product by BI in 2021 with the intention of bringing it to a first-in-man trial in CF patients.

##### Herpes Simplex Virus 1: HSV-1

Krystal Biotech have engineered a replication-defective HSV-1 gene therapy vector product (KB407) encoding two copies of full-length human CFTR for the treatment of CF. Reports from conference proceedings suggest that KB407 possesses a robust safety and efficacy profile in multiple in vitro CF models including functional restoration of CFTR in clinically relevant patient-derived intestinal organoids cultured from four CF patients^65,67^. Administration of inhaled KB407 to NHPs on three weekly occasions was well tolerated and gave widespread expression in the lung until at least 28 days after the third dose^67^. Although there have as yet been no significant peer-reviewed publications that verify these outcomes, patients are currently being recruited for a Phase 1 trial in Australia and the FDA have granted approval for a Phase 1 trial in CF in the US. https://ir.krystalbio.com/news-releases/news-release-details/krystal-biotech-announces-fda-acceptance-kb407-ind-application.

###### Non-Viral

The advantages of non-viral systems described above led to a wave of CF gene therapy clinical trials with such vectors in the mid 1990s to early 2000s^14^: however, the transient, low level expression observed led to a refinement of non-viral strategies. Rather than focusing only on improving the gene transfer agent, therapeutic plasmids that allowed greater persistence of expression were developed. A move away from strong viral promoters such as the CMVie promoter to cell-derived alternatives such as the human elongation factor 1 alpha promoter or ubiquitin B or C promoter showed that extended duration was possible^95^. When these were used in the context of CpG dinucleotide depleted constructs^96^, the inflammatory response to unmethylated CpGs in the bacterially-sourced plasmid DNA was reduced, and persistence of expression was even more pronounced. These advances were incorporated into a formulation of the improved plasmid pGM169 with the cationic liposome GL67A, which was evaluated in two clinical trials. A single dose pilot study that showed changes in CFTR-mediated chloride transport^97^ was followed by a monthly repeat dose trial over 12 months: this achieved its primary clinical outcome, namely significant but modest stabilization of lung function, as measured by forced expiratory volume in 1s (FEV1), while the lung function of placebo recipients declined over time^71^. Despite this encouraging clinical proof-of-concept, the magnitude of the effect was deemed insufficient to justify proceeding immediately to Phase III studies as a stand-alone therapy, particularly in the context of the lung function improvements observed with modulator therapies.

###### RNA-based

Interest in non-viral approaches has also been revived as a method for delivery of RNA-based therapeutics for CF. Examples include mRNA formulations expressing the CFTR protein, and antisense oligonucleotides targeted at mutant *CFTR* transcripts to encourage mRNA repair permitting transcript readthrough or correcting CFTR splice mutations. Importantly, unlike the transfer of DNA cargo, RNA does not require nuclear localization or transcription. An additional benefit is the negligible risk of genomic integration of the delivered sequence. On the other hand, a disadvantage of this approach ist that delivery of mRNA normally results in a short duration of expression. A number of strategies to chemically modify mRNA for enhanced stability have been explored to improve duration^98^. An alternative is to complex mRNA with lipid nanoparticles. This approach was taken by Translate Bio who recently reported on their first-in-man Phase I/II trial of repeat delivery of MRT5005, a formulation of fully functional CFTR mRNA with lipid nanoparticles https://www.biospace.com/article/releases/translate-bio-presents-mrt5005-data-at-the-33rd-annual-north-american-cystic-fibrosis-conference/. Disappointingly, this did not significantly improve lung function in patients despite promising data from an earlier single dose trial^99^.

Eluforsen (formerly QR-010) is an antisense oligonucleotide binding the mRNA region encoding the Phe508del CFTR mutation, and was developed clinically by ProQR Therapeutics for inhalation to patients with F508del CF. The Phase I clinical trial (NCT02564354) initiated in 2015 showed that repeated administration to the nasal epithelia (three times per week for 4 weeks) was well tolerated with a favourable safety profile and that CFTR activity was restored in the homozygous Phe508del CF patient cohort (n=7) after intranasal administration, as determined by improved total chloride transport measured by nasal potential difference (NPD). Improved CFTR function was not observed in the compound heterozygous cohort^100^. A dose-escalation Phase Ib clinical trial (NCT02532764) in homozygous Phe508del CF patients was completed and demonstrated that single and multiple doses of inhaled Eluforsen were safe and well tolerated^101^. However, as a result of the lack of significant effect on lung function or sweat chloride, further clinical development of this candidate was discontinued.

##### Gene Editing

An alternative non-viral gene editing strategy being evaluated as a therapeutic for CF uses cell penetrating peptides (CPPs) that can be linked to ribonucleoproteins (RNPs) comprising Cas protein and gRNA. CPPs are generally 5 to 30 amino acids and whether cationic, nonpolar or amphipathic share the ability to rapidly cross the cell membrane and mediate uptake to cells including cultured human ciliated and non-ciliated epithelial cells and mouse airway epithelia in vivo^80^. Delivery of BE loaded RNP linked to amphiphilic CPPs into the airways of rhesus monkeys to target airway epithelial cells achieved in vivo editing at the CCR5 locus with up to 5.3% efficiency, a level considered therapeutically relevant in CF^102^.

#### ALPHA-1-Antitrypsin Deficiency : AATD

Mutations in the SERPINA 1 gene lead to low levels of alpha-1 antitrypsin (AAT) in the lung interstitium. AAT is a serine protease inhibitor that inhibits neutrophil elastase and other serine proteases (OMIM #613490). In patients with AAT deficiency the protease/anti-protease balance in the lung is perturbed and leaves the lung parenchyma vulnerable to protease mediated damage resulting emphysema, chronic bronchiolitis and ultimately declining lung function^106^. AAT is primarily produced in the liver and reaches the lung by diffusion from the circulation although small amounts are also produced locally in the lung from bronchial epithelial cells and mononuclear phagocytes. Augmentation with purified AAT protein has been introduced in some countries but has limited efficacy in slowing the decline in lung function, requires regular intravenous infusion and is very costly^107^. An effective gene therapy strategy, non-invasively applied to the lungs, could lead to long-lasting expression of AAT in airways and alveoli from a single administration. It should also provide stable levels of AAT rather than the peaks and troughs achieved with protein infusions. AAT expression in alveoli will directly protect the interstitium from elastase degradation, whereas expression of AAT in the airways will reduce the neutrophil chemo-attractants interleukin 8 and leukotriene B4 31, thereby reducing neutrophil infiltration and lowering the burden of neutrophil elastase. Adjusting the imbalanced protease/antiprotease ratio may lead to therapeutic benefit in AAT deficient patients, as well as patients with other inflammatory lung diseases.

##### Gene Therapy for AATD

As with CF, gene therapy approaches for AATD date back several decades. Preclinical studies with retroviral or Ad vectors failed to show sufficient promise to progress to clinical studies due to limitations of the vector systems^108^. Initial clinical studies included non-viral plasmid-liposome to a single nostril^109^ and provided evidence of transient expression in nasal lavage and concurrent anti-inflammatory activity that had returned to baseline by day 14. rAAV clinical trials delivering AAV1 /AAV2 vectors via intramuscular delivery failed to achieve therapeutic levels even in a Phase II study with higher doses of vector^108,110–112^. This turned attention to direct administration to the lung but despite significant effort by several groups, with a variety of AAV serotypes selected for lung efficacy, the levels of AAT achieved in preclinical studies did not warrant progress to clinical trials. An alternative route to achieving AAT expression in the lung is through delivery to the intrapleural space^6^. This approach relies on the AAT produced by transduced mesothelial cells lining the pleura firstly being secreted to the lung interstitium via the basolateral surface to give local AAT and secondly being delivered systemically by vector reaching the liver via the visceral lymphatics. An NHP-derived serotype AAVrh.10 was identified as the most effective at generating sustained high expression of normal human M-type AAT in serum and also lung epithelial lining fluid mice^113^. A subsequent safety & efficacy study in NHPs^114^ demonstrated high hAAT mRNA expression for the 1 year duration of the study. The data from the ADVANCE Phase 1/2 study of ADVM-043 (AAVrh.lOhAAT), did not reach threshold levels of 11μM in the treated patients and Adverum Biotechnologies, who licensed the AAVrh.10hAAT technology, announced its decision to discontinue the development of ADVM-043.

Two other approaches for AATD gene therapy are currently ongoing. One uses the rSIV.F/HN lentiviral vector platform discussed above for CF. Preclinical studies have shown that the rSIV.F/HN vector can transduce all of the epithelial cell types in the lung and can deliver transgenes to both the conducting airways and the gas exchanging alveolar regions^45^. Preclinical studies in mice were performed by intranasal delivery of the vector rSIV.F/HN-hCEFsohAAT expressing hAATcDNA driven by an hCEF promoter^115^. Sustained expression of hAAT could be measured both in lung tissue homogenates and in ELF and importantly, hAAT expression reached the therapeutic target of ~ 11μM in epithelial lining fluid (ELF) of mice for the duration of the study (19 months). hAAT could also be detected in serum of these animals and levels were highly correlated with levels in ELF suggesting that serum levels may be predictive of lung levels and could provide a non-invasive assay for longitudinal monitoring of lung hAAT in clinical studies.

An HSV-1 vector (KB408) is also under development for AATD by Krystal Biotech using the vector platform described for their CF program above. KB408 can efficiently transduce clinically relevant primary human small airway epithelia cells, resulting in production and secretion of full-length human AAT^66^. Inhaled KB408 also effectively targeted the respiratory tract of mice resulting in detectable levels of human AAT in both the serum and lung lining fluid. On the basis of these data Krystal Biotech has submitted a pre-IND (Investigational New Drug) briefing package and received approval from the FDA for a Phase 1 study of KB408 in patients in Australia and the US (https://ir.krystalbio.com/news-releases/news-release-details/krystal-biotech-provides-updates-its-rare-genetic-lung-disease). Finally, gene editing approaches are also under development for AATD. Beam Therapeutics is in preclinical development of CRISPR base editing strategies that correct the most common pathological AAT allele, PiZ^116^. In NOD scid gamma (NSG) PiZ knock-in mice, intravenous injection of lipid nanoparticle vectors carrying mRNA encoding BEs decreased markers of liver pathogenesis and increased serum AAT levels. Additionally, Intellia Therapeutics are investigating a “Remove / Restore” strategy using CRISPR/Cas9 to knock-out pathological AAT alleles and insert functional copies of AAT in the albumin locus^117^. In NHPs, this approach leads to durable AAT production nearing the therapeutic threshold levels seen in people with non-pathogenic alleles. Although neither of these approaches directly target the lung the elevated serum levels of AAT should ameliorate lung disease.

#### Surfactant Deficiencies

Pulmonary surfactant is produced in alveolar type 2 (AT2) cells and is a complex mixture of phospholipids and proteins that functions to reduce surface tension at the alveoli air–liquid interface. The protein component of surfactant comprises 4 major proteins, namely, surfactant proteins SP-A, SP-B, SP-C and SP-D. The adenosine triphosphate (ATP) binding cassette 3 (ABCA3) protein is also critical in surfactant production as it facilitates transport of phospholipids into intracellular surfactant storage vesicles termed lamellar bodies where they assemble with SP-B and SP-C to form surfactant prior to exocytosis^120^. Mutations in genes *SFTPB* and *SFTPC*, encoding SP-B and SP-C respectively, as well as mutations in the *ABCA3* gene cause surfactant deficiency and are leading inherited causes of childhood interstitial lung disease (ILD)^121^. Mutations in both *SFTPB* and *ABCA3* exhibit loss of function phenotypes. SPB deficiency is a rare (approximately 1 birth per million^120^) autosomal recessive condition which carries a very poor prognosis with mortality within the first few months of birth. Synthetic surfactant formulations that significantly improve the prognosis of pre-term newborns are ineffective. The only potentially effective treatment currently available is lung transplantation^122^, although with donor availability for paediatric lung transplants so limited it is rarely attempted in these patients. Mutations in *ABCA3* are the most common cause of genetic surfactant dysfunction occurring approximately five times more frequently than SP-B deficiency. ABCA3 deficiency is also an autosomal recessive condition causing ILD but with a more variable clinical course depending on the mutation. ^121,123^. There is no single common *ABCA3* mutation, however subjects with two null mutations are typically severely affected and have a similar clinical outcome to SP-B deficiency. A clear opportunity exists to develop and introduce a new therapeutic addressing the significant unmet medical need for patients with these surfactant deficient conditions.

##### Gene Therapy for Surfactant deficiencies

Efforts to develop gene therapy approaches for SP-B deficiency were first reported in 1994. The Ad type 5 (Ad5)-based Av1SP-B1, in which the full length human SP-B cDNA is expressed under the control of the Rous sarcoma virus (RSV) promoter in an E1-E3-deleted Ad type 5 (Ad5)-based vector system, was delivered to the lungs of cotton rats^124^. Human SP-B peptide was detected as secreted, 8kD and 18kD monomeric and dimeric forms, suggesting that the vector-derived precursor SP-B (proSP-B) was appropriately processed after in vivo gene transfer thus providing proof-of principle. As discussed above for other respiratory diseases, the use of Ad vectors for gene delivery to the lung has been largely abandoned due to poor receptor availability, immune responses and a short-lived expression profile. A different strategy used chemically modified mRNA replacing approximately 25% of uridine and cytidine residues with 2-thiouridine and 5-methyl-cytidine to increase stability and decrease host inflammatory responses. In a mouse model of SP-B deficiency, twice weekly local application of an aerosol of chemically modified SP-B mRNA to the lung for 4 weeks restored 71% of the wild-type SP-B expression^125^, with treated mice surviving until the predetermined end of the study after 28 days. This technology is now embedded in the company Ethris who have a pipeline for pulmonary indications other than SP-B deficiency https://www.ethris.com/pipeline/. The same mouse model of SP-B deficiency was used to evaluate a physical gene therapy approach using electroporation to deliver plasmid DNA expressing SP-B cDNA^126^. Increased survival and improvements in lamellar body morphology once again provided proof-of-concept although a translational path for use of such an electroporation-based strategy seems challenging^127^. Proof-of-principle has also been provided by studies using patient-derived iPSCs from a patient with SP-B deficiency; transduction with a lentiviral vector expressing SP-B and subsequent differentiation of the iPSCs into organoids resulted in functional mature lamellar bodies and secretion of SP-B into the supernatant^128^. More recently the SP-B deficient mouse model was used to validate a viral vector-mediated approach using AAV6.2FF serotype, an AAV capsid containing an amino acid substitution that facilitates heparin binding, abrogates ubiquitin-mediated degradation and targets cells highly expressing the cell surface epithelial cell adhesion molecule (EpCAM). Intratracheal delivery of the vector resulted in prolonged survival for over 200 days in some cases^129^. Interestingly the therapeutic effect was enhanced by co-administration of bovine pulmonary surfactant which may have aided distribution of the vector throughout the murine lung. Aside from the SP-B deficient mouse model which is useful for benchmarking in vivo gene delivery strategies, a new human model of ATII cells has been developed. The surfactant air-liquid interface (SALI) culture model is based on human H441 cells in which the *SFTPB* gene has been knocked out by gene editing^130^ and shows a gene expression profile similar to ATII cells when analysed by single-cell RNA-sequencing^131^. The SALI culture model successfully recapitulates the key characteristics of human ATII cells in primary culture and when the *SFTPB* KO cells exhibited altered functional barrier properties (reduced transepithelial electrical resistance) this could be corrected by delivery of rSIV.F/HN-expressing SPB protein^130^.

### Pulmonary Alveolar Proteinosis

Pulmonary alveolar proteinosis (PAP) is a rare, life-limiting lung disease characterized by accumulation of surfactant in the alveoli leading to infections, lung fibrosis, and, ultimately, respiratory failure. Clearance of pulmonary surfactant is mediated primarily by ATII cells, which take up approximately 70-80% of surfactant for recycling or degradation. The remaining 20-30% is mostly phagocytized and catabolized by alveolar macrophages and granulocyte-macrophage colony-stimulating factor (GM-CSF) is required for this process^132,133^. About 90% of PAP cases are caused by generation of anti-GM-CSF autoantibodies (aPAP), which prevent adequate GM-CSF-mediated surfactant clearance by alveolar macrophages^134,135^. Patients require regular whole-lung lavage under anaesthesia, to wash out the excess lipoproteinaceous surfactant. The lavage procedure is associated with complications and is only performed in specialist centres^136^. Inhaled recombinant GM-CSF protein therapy approaches have shown some benefit in a Phase 2 trial (IMPALA) although the effects are modest with uninterrupted daily administrations, probably due to the short half-life of the protein^137^. (IMPALA-2 Phase 3 trial is currently underway: NCT04544293). An effective gene therapy has the potential to provide less frequent dosing, and a more stable steady-state GM-CSF concentration profile, as the therapeutic protein will be produced locally where required. Hereditary PAP (herPAP) accounts for 5% of cases and is caused by recessive mutations in the cell surface receptors which mediate binding of GM-CSF on myeloid cells^138^.

#### Gene therapy for PAP

Gene therapy strategies for aPAP can be evaluated in vivo using GM-CSF knockout mice where surfactant synthesis and secretion occur at a normal rate, but recycling and/or clearance are impaired^139^. In the earliest studies in these mice, intratracheal administration of an Ad vector expressing GM-CSF led to transient (1–3 weeks) low levels of GM-CSF expression, which corrected some biomarkers of the disease but only when the mice were transiently immunosuppressed^140^. However, it has been well documented that Ad vectors induce immune responses, lose efficacy when administered repeatedly and are therefore un-likely to be suitable for treatment of chronic lung diseases. Recent studies with the rSIV.F/HN lentiviral vector platform described in previous sections assessed efficacy of rSIV.F/HN-mediated gene transfer in the GM-CSF knockout mice following a single dose of rSIV.F/HN expressing murine (m)GM-CSF (1e5-92e7 TU/mouse).

Expression of mGM-CSF was dose-related and persisted for the duration of the experiment (∼11 months). Biomarkers of PAP disease were rapidly and persistently ameliorated. Long term expression of high levels of GM-CSF caused histopathological changes in various organs at the highest doses, but correction of some PAP biomarkers was also achieved with very low doses of vector (1e5 TU/mouse), which did not induce histopathological changes over an 11 month study period^141^. This study provides evidence for long term expression of a secreted transgene providing functional benefit in a mouse model but also indicates that there is a balance between efficacy and toxicity that will require consideration for translation to the clinic. Recent publications from the Trapnell group demonstrate an alternative cell therapy approach for treating herPAP. In mouse models of herPAP, intrapulmonary transplantation of wild-type or gene-corrected bone marrow-derived or iPSC-derived macrophages showed long term residence of transplanted cells and improvements in alveolar protein deposition and other critical herPAP disease parameters^142–144^ indicating the potential for cell-based therapeutic options. Initial work demonstrating a similar approach to treat the autoimmune form of PAP via macrophage transplantation, where transplanted cells are genetically modified to express GM-CSF has also recently been reported in conference proceedings^145^.

### Vectored Immunoprophylaxis (VIP)

Another interesting application of gene therapy vectors in the lung is vectored immunoprophylaxis (VIP) to provide passive immunity against infectious disease^146^. The original papers described VIP against HIV infection with AAV2/8 vectors expressing broadly neutralising anti-HIV antibody VRC07 systemically following intramuscular (IM) injection. This approach has gone as far as Phase 1 clinical trial in adults living with HIV^147–149^. The same research group also demonstrated t IM delivery of AAV vectors expressing broadly neutralizing antibodies against influenza protected mice against challenge with diverse influenza strains resulting in only minimal weight loss and inflammation^150^. The authors speculated that if translated successfully to humans, this could provide a unique route for protecting immunocompromised or elderly patient populations for whom existing flu vaccines are ineffective. This was taken a step further when AAV9 vector expressing a modified version of antibody FI6, a broadly neutralizing mAb to influenza A, was delivered intranasally (IN) to both mice and ferrets and showed complete protection against challenge with clinical isolates of human pandemic influenza^151^. Expression of F16 from AAV9 vector was capable of offering some protection against influenza challenge as early as 3 days after administration. A similar IN delivery of AAV9 vector to express a combination of antibodies, derived from the Ebola treatment ZMapp, protected mice from airway challenge against mouse-adapted Ebola virus strain MA-ZEBOV^152^. Similarly, IN delivery of recombinant AAV9 expressing either the FDA-approved anti-RSV antibody palivizumab or the more potent second generation version motavizumab, significantly reduced the RSV load in the lungs of a BALB/c mouse model of infection^153^. Serum-circulating neutralizing AAV9 antibodies induced by a previous dose of AAV9 vector expressing an irrelevant reporter gene had no impact on the ability of AAV9 vector expressing palivizumab to reduce RSV load in the lung after challenge. If this observation translates to human studies it would imply that repeated administration of AAV9 vector to the airway for seasonal prophylaxis against RSV may be possible, thereby expanding the relevance of the strategy against various airborne viruses. A similar strategy of using a lentiviral vector platform also achieved passive immunity in the lung against influenza^154^,SARS-COV2^155^ and RSV^156^. The recombinant SIV vector pseudotyped with Sendai virus F/HN envelope proteins (rSIV.F/HN; see above), was used to deliver the neutralising anti-influenza monoclonal antibody (nAb) T1-3B^157^ and generated levels of antibody in the lung lumen and serum sufficient to protect mice from a lethal dose of two diverse influenza strains with protection levels equivalent to that obtained with an AAV9 vector expressing the same nAb. When the same rSIV.F/HN vector platform was used to deliver anti-SARS-CoV-2 RBD nAb, NC0321, it efficiently neutralised a range of SARS-CoV-2 variants, including Alpha, Beta, Delta and Eta. Expression of NC0321 nAb conferred protection from infection with a SARS-CoV-2 mimic in a mouse model expressing human ACE2^155^ and, importantly, demonstrated that aged or immunodeficient mice could also be protected from challenge with a mouse adapted SARS-CoV-2 viral mimic^158^. This highlights the potential for such a strategy to offer protective immunity in vulnerable populations. Vector-mediated protection in vivo was also conferred against RSV challenge using the rSIV.F/HN vector platform expressing palivizumab, offering complete protection from RSV-induced weight loss and a trend towards an improved lung leukocyte recruitment profile (an indicator of RSV-induced inflammation)^156^. It is worth noting that both the rSIV.F/HN (IN) and AAV8 (IM) vectors-tested provided sufficient protection, although higher palivizumab levels were achieved in the serum and lung lumen of mice when using AAV. As discussed above, however, the lentiviral platform offers a greater capacity for the packaging of large transgenes and the ability to be repeatedly administered should prophylaxis wane over time. Overall, the data from these studies suggest that VIP could be used to induce immunity in individuals for whom conventional vaccines lack efficacy; such an approach may also be of value in protecting health workers and other essential personnel for prolonged periods during emerging pandemics.

### Idiopathic Pulmonary Fibrosis

Idiopathic pulmonary fibrosis (IPF) is a chronic and progressive lung disease resulting from pathological deposition of extracellular matrix (ECM) in the lungs. A variety of mechanisms have been implicated in the disease process: the consensus is that the process is largely driven by dysregulated and persistent tissue repair processes that lead to a gradual decline in lung function and ultimately respiratory failure^159^. The prognosis for sufferers is poor with median survival of 2-3 years from diagnosis^160^. The pathophysiology of IPF is complex and not fully understood, although chronic injury of alveolar epithelial type II (ATII) cells is an essential hallmark of the disease and dysregulates regenerative capacity and epithelial-mesenchymal interactions^161^. There is increasing global mortality from IPF: risk factors such as smoking, air pollution, chronic respiratory viral infections and, in a few cases, genetic predisposition have been identified^162^. The only two currently approved therapeutic options are the antifibrotic agents pirfenidone and nintedanib, which slow the progression of IPF but are not curative.

#### Gene therapy for IPF

The most challenging issue for IPF gene therapy development is the identification of suitable targets, since IPF has a complex aetiology and is not a monogenic disease. Another significant issue is the lack of a good animal model capable of accurately reflecting all disease features of IPF^163^. A number of gene therapy strategies have been evaluated in model systems, with vectors expressing a wide range of candidate transgenes, the majority of which were prophylactic approaches using Ad vectors^164^. Here we describe two gene therapy approaches which have demonstrated some efficacy in models with established fibrosis, the situation which exists in IPF patients at diagnosis.

Some familial and sporadic patients with IPF have shortened telomeres, which is hypothesised to reduce cellular repair capacity. AAV9 vectors were used to investigate the efficacy of telomerase reverse transcriptase (TERT) gene transfer in the low-dose bleomycin (BLM) induced IPF model in telomerase deficient mice^165^. Intravenous delivery of rAAV9.TERT predominantly transduced ATII cells. Importantly, TERT gene transfer decreased inflammation, inhibited fibrosis 1-3 weeks after delivery and even partially reversed established fibrosis. A transcriptome analysis of TERT-transduced ATII cells confirmed the inhibition of fibrosis and inflammation pathways, and ATII cell proliferation was increased. More recently the same group^166^ reported ageing-associated development of shortened telomeres, decreased numbers of ATII cells, impaired surfactant activity and a profibrotic phenotype in wild type mice: all features concordant with observations made in telomerase-deficient mice which could be prevented in both wild type and telomerase-deficient mice following delivery of AAV9 vector expressing TERT. Telomere Therapeutics was formed in 2020 to support clinical development of this approach https://www.cnio.es/en/news/cnio-news/spin-off-company-first-treatment-against-pulmonary-fibrosis-based-on-telomerase-gene-therapy/.

Another recent approach has been to target calcium homeostasis and impair myofibroblast differentiation and function, based on the observation that a calcium channel blocker inhibited BLM-induced IPF in mice by decreasing ECM deposition, soluble collagen and hydroxyproline levels. Additionally, a decrease in SERCA2a levels was noted in IPF patients and BLM-challenged mice^167^. Intratracheal nebulization of AAV1 vector expressing SERCA2a effectively reduced lung fibrosis and vascular remodelling, improved gas exchange and increased the lifespan by 45% compared to BLM mice treated with a control AAV encoding luciferase^167,168^. Interestingly the effects of AAV1 vector mediated expression ofSERCA2a on BLM-induced fibrosis were also observed when administered two weeks prior to BLM treatment in a prophylactic manner.

These approaches all involve in gene addition/enhancement strategies: however, the review by Ruigrok^164^ also discusses strategies which manipulate expression of a variety of genes involved in inflammatory or profibrotic processes including myofibroblast differentiation, ECM synthesis, epithelial-mesenchymal transition, through delivery of siRNA or miRNA. Again, the vast majority of these utilise a prophylactic approach in preventing BLM-induced injury: although some efficacy was observed in most studies, the relevance of this to IPF patients who present with already established fibrosis, and in whom the contribution of inflammation is not straightforward, would suggest that further pre-clinical validation will be necessary in advance of clinical studies.

We also note that vectors expressing combinations of siRNA, miRNA and transgenes to synergistically manipulate expression in multiple pathways could be a productive strategy in the development of gene therapies for a multifactorial disease such as IPF where there is no obvious single target. A similar argument suggests that combinations of gene and small molecule therapies may also provide improved efficacy against IPF.

## Conclusions

Significant advances have been made in gene therapeutic approaches for respiratory diseases where there are clearly identified targets. For gene addition strategies there will continue to be design innovations across a range of vector types aimed at improving efficacy and safety. Further improvements in targeting relevant cell types either through physical targeting of vectors, by delivery or by regulation of expression of transgenes using cell-specific promoters will all likely have a role to play. The rapid innovation in gene editing technology has the potential to make a significant contribution to clinical gene therapy. However, the overall low efficiency and specificity of gene editing in certain target tissues such as mature airway epithelium in vivo challenge its use and pose safety concerns. Delivery of the editing machinery still remains one of the biggest issues and this may be even more of a rate-limiting step for the potentially safer but larger prime editing machinery. Similarly, until gene addition with integrating vectors or genome editing can be made to efficiently target resident stem or progenitor cells in the lung, the additional challenge of repeated administration and potential immune responses will remain a significant consideration.

For non-genetic diseases where the targets are less clear, advances in genomics, transcriptomics and techniques such as single cell sequencing are helping to elucidate the pathways involved in disease pathogenesis and will allow rational development of more sophisticated therapeutic approaches based on regulating disease-causing pathways and mechanisms.

In light of the significant and continuing progress in gene therapy, with increasingly more products receiving market authorization (https://www.biocentury.com/article/644637), there are genuine grounds for optimism that gene therapy for a range of respiratory diseases will enter clinical practice.

## Figures and Tables

**Table 1:** Features of viral vectors currently being evaluated for respiratory gene therapy.

Viral Vectors				
	Features	Integrating	Advantages	Disadvantages
**AAV**				
Multiple serotypes	Altered tropism by directed evolution, capsid shuffling, peptide display. Dual AAV. Parvovirus Chimeras	Yes, but at lower frequency than integration-requiring vectors	Low Immunogenicity Persistent expression	Limited packaging capacity. Pre-existing or induced neutralising antibodies. Unable to repeat administer. Low risk of insertional mutagenesis, Hepatotoxicity at high dose
**LV**				
Backbone HIV, FIV, SIV	Self inactivating. Pseudotyping to alter tropism eg VSV-G, GP64 Sendai F/HN	Yes	Low immunogenicity. Persistent expression. Large packaging capacity. Repeat Administration	Risk of insertional mutagenesis.
**HD-Ad**				
Multiple serotypes	Limited viral protein expression.	No	Low immunogenicity. Very large packaging capacity	Multiple repeat administration unlikely
**HSV-1**				
	Unknown. Krystal Biotech Proprietary vector	No	Low immunogenicity. Very large packaging capacity. Repeat administration	Limited peer-reviewed data to date

AAV, Adeno Associated Virus; LV, Lentivirus; HD-Ad, Helper-Dependent Adenovirus, HSV-1, Herpes Simplex Virus 1.

**Table 2:** Recent gene therapy approaches for CF lung disease. ITR, Inverted Terminal Repeat; CMV-ie, Cytomegalovirus immediate early enhancer/ promoter; RSV, Rous sarcoma virus; hCEF, Elongation factor 1α promoter with human CMV enhancer.

CF					
Vector	Promoter	CFTR	Features/ Status	Repeat Admin	Refs
AAV2 1^st^ Generation	AAV ITR	Full length	Multiple Trials: Low efficacy in clinic:	No	14,28,86
AAV2.5T (SP101) Spirovant	Shortened CMV-IE promoter	N-terminal deletion of R domain	Alternative receptor-Sialic Acid Preclinical development.	No	17,88
Variant A101 (4D-710) 4D Molecular Therapeutics	Proprietary	N-terminal deletion of R domain	AAV capsid variant library “Therapeutic Vector Evolution”) in NHP. Phase 1/2	Not known. Exhibited resistance to pre-existing human antibodies in IVIG in vitro even at high titres (1:50), compared to wild-type serotypes	18,23
rFIV GP64 (SP-102) rHIV-GP64	RSV	Full length	Baculovirus GP64 confers apical tropism. Preclinical development.	Yes	103–105
rSIV F/HN UK Resp GeneTher Consortium/ Boehringer Ingelheim	hCEF hybrid promoter.	Full length	Sendai Virus F/HN confers apical tropism. Preclinical development.	Yes	44,45
HSV1 (KB407) Krystal Biotech	Unknown	Full length	Apical tropism. Phase 1 recruiting	Possibly	65–67
Plasmid DNA/Cationic liposome pGM169/GL6 7A	hCEF hybrid promoter.	Full Length	Phase 1/2b Clinical Trials completed Stabilisation of Lung function	Yes	97
mRNA plus lipid nanoparticles MRT5005, Translate Bio	N/A	biosynthetic mRNA coding for CFTR	Phase I/II completed. Low efficacy	Yes	99 https://www.biopharmadive.com/news/translate-bio-mrna-cystic-fibrosisnegative-results/596930/

**Table 3:** Recent gene therapy approaches for AATD. CMV, Cytomegalovirus immediate early enhancer/promoter; ITR, Inverted Terminal Repeat; CAG, CMV enhancer fused to the chicken betaactin promoter; hCEF, Elongation factor 1α promoter with human CMV enhancer.

AATD				
Vector	Route of delivery	Promoter	Status	Refs
Plasmid Liposome pCMV-AAT DOTMA/ DOPE	Nasal epithelium	CMV	Phase 1	109
AAV1 & AAV2	Intramuscular	CAG	Phase 1/2	110,118,119,111,112
AAV5/AAV rh.10 Adverum	Intrapleural	CAG	ADVANCE Phase 1/2	114 https://investors.adverum.com/news/news-details/2018/Adverum-Biotechnologies-Provides-Program-Updates-2018-11-1-2018-11-1/default.aspx
rSIV.F/HN (hCEFsohAAT)	Pulmonary	hCEF	Pre-Clinical	115
HSV1 KB408 Krystal Biotech	Pulmonary		Pre-Clinical	66 https://ir.krystalbio.com/news-releases/news-release-details/krystal-biotech-announces-virtual-presentation-pre-clinical-0
Lipid nanoparticles	Intravenous	N/A	Pre-Clinical	116
Lipid nanoparticles and rAAV	Intravenous	N/A	Pre-Clinical	117

**Table 4:** Recent gene therapy approaches for surfactant deficiencies. UbC, Human Ubiquitin C; CMV, Cytomegalovirus immediate early enhancer/promoter; CASI, human CMVie enhancer region, the chicken beta actin promoter, and the human ubiquitin C promoter; hCEF, Elongation factor 1α promoter with human CMV enhancer.

Surfactant deficiencies					
Vector	Promoter	Transgene	Model	Status	Ref
Chemically modified RNA	N/A	Human proSPB	SPB deficient mouse model	Discontinued (?)	125
Plasmid DNA electroporation	UbC, CMV	Human proSPB	SPB deficient mouse model	Preclinical	126
Lentivirus, HIV. VSV-G pseudotyped	CMV	Human proSPB	Patient iPSC-derived alveospheres	Preclinical	128
AAV6.2FF	Composite CASI promoter	Human proSPB	SPB deficient mouse model	Preclinical	129
Lentivirus rSIV.F/HN	hCEF	Human proSPB	SPB KO human SALI model	Preclinical	130

**Table 5:** Recent gene therapy approaches for Pulmonary Alveolar Proteinosis. RSV, Rous sarcoma virus; hCEF, Elongation factor 1α promoter with human CMV enhancer; EFS, Short elongation factor 1 promoter. mGM-CSF, murine granulocyte-macrophage colony-stimulating factor; mCsf2ra, murine colony-stimulating factor 2 receptor alpha.

Pulmonary Aleolar Proteinosis					
Vector	Promoter	Transgene	Model	Target	Ref
AV1mGM (Ad 5 serotype)	RSV	mGM-CSF	GM-CSF knockout mice (aPAP)	Lung	140
rSIV.F/HN-GM-CSF	hCEF	mGM-CSF	GM-CSF knockout mice (aPAP)	Lung	141
mCsf2ra-LV	EFS	mCsf2ra	Csf2ra-/-mice	Ex vivo HSPC-derived macrophages (Cell therapy)	144

**Table 6:** Recent gene therapy approaches for vectored immunoprophylaxis. CAG, CMV enhancer fused to the chicken beta-actin promoter; CB7, chicken β-actin promoter with cytomegalovirus enhancer elements ; hCEF, Elongation factor 1α promoter with human CMV enhancer; CASI, human CMVie enhancer region, the chicken beta actin promoter, and the human ubiquitin C promoter.

Vectored Immuno Prophylaxis					
Vector	Antibody	Promoter	Delivery/Dose	Challenge Virus	Ref
AAV9	F16 (AAV9.F16)	CAG	IN Mice @ 3e10 to 1e11 GC Ferrets @ 1e12 GC	Multiple clinical isolates of influenza including pandemic strains	151
AAV9	2 ZMapp Ab components (AAV.2G4, AAV.c13C6)	CB7	IN or IM Mice @ 1e11 GC	Mouse-adapted Zaire Ebola Virus	152
AAV9	Palivizumab (AAV9.Pal-IA) Motavizumab (AAV9.Mot-IA)	CAG	IN Mice @ 1e9 to 1e11 GC. Decreased viral load	RSV strain A2 (VR-1540; ATCC, Manassas, VA)	153
rSIV.F/HN AAV9 AAV8	T1-3B (rSIV.F/HN.T13B) (rAAV2/9.T13B) (rAAV2/8.T13B)	hCEF hCEF CASI	IN Mice @ 1e8 or 2.7e8 TU Mice @ 1e11 GC (IM AAV8)	PR8 strain H1N1 A/Puerto Rico/8/1934 or reassortant pandemic H1N1 A/CA/7/2009-X179A	154
rSIV.F/HN AAV9 AAV8	NC0321 (rSIV.F/HN.NC0321) (rAAV9.NC0321) (rAAV8.NC0321)	hCEF hCEF CASI	IN Mice @ 1e8 or 2.7e8 TU Mice @ 1e11 GC (IM AAV8)	SARS-CoV-2 mimics or Authentic SARS-CoV-2 virus	155 158
rSIV.F/HN AAV8	Palivizumab (rSIV.F/HN.Palivizumab) (rAAV8. Palivizumab)	hCEF CASI	IN Mice @ 1e6, 1e7, 1e8 or 2e8 TU IM Mice @ 1e9, 1e10, or 1e11 GC	RSV strain A2 (VR-1540; ATCC, Manassas, VA)	156

**Table 7:** Recent completed or ongoing clinical trials of nucleic acid delivery for respiratory disease. Trial details can be found using NCT Identifier number at https://clinicaltrials.gov/. CF, Cystic Fibrosis; AATD, Alpha 1 Antitrypsin Deficiency; RSV, Rous sarcoma virus. hCEF, Elongation factor 1α promoter with human cytomegalovirus (CMV) enhancer; CAG, CMV enhancer fused to the chicken beta-actin promoter.

Clinical Trials					
	Disease	Product	Details	Promoter	ClinicalTrials.gov Identifier
UK CFGene Therapy Consortium	CF	pGM169/GL67 A	Plasmid DNA/Cationic liposome	hCEF	NCT01621867
Adverum	AATD	ADVM-043	AAVrh.10halpha1A T	CAG	NCT02168686 NCT03804021
Translate Bio	CF	MRT5005,	Inhaled mRNA therapeutic	N/A	NCT03375047
4D Molecular Therapeutics	CF	4D-710	A101 variant	Proprietary	NCT05248230
ProQR Therapeutics	CF	Eluforsen (formerly QR-010	Antisense oligonucleotide binding the mRNA region encoding the Phe508del CFTR mutation	N/A	NCT 02564354 NCT 02532764
Krystal Biotech	CF	KB407	HSV-1	Proprietary	NCT05095246 NCT05504837
Alnylam	RSV	ALN-RSV01	siRNA targeting the RSV nucleocapsid protein	N/A	NCT00496821, NCT00658086, and NCT01065935)
